# Ultrafast Blood T_1_ Measurement Using Golden Angle Rotated Spiral k‐t Sparse Parallel Imaging (GASSP): Evaluations in Both Pre‐ and Post‐Contrast Conditions

**DOI:** 10.1002/mrm.70286

**Published:** 2026-02-06

**Authors:** Zechen Xu, Feng Xu, Qin Qin, Dan Zhu

**Affiliations:** ^1^ Department of Biomedical Engineering Johns Hopkins University School of Medicine Baltimore Maryland USA; ^2^ F.M. Kirby Research Center for Functional Brain Imaging, Kennedy Krieger Institute Baltimore Maryland USA; ^3^ The Russell H. Morgan Department of Radiology and Radiological Science Johns Hopkins University School of Medicine Baltimore Maryland USA; ^4^ Diagnostic Imaging Sciences Center, Department of Radiology University of Washington School of Medicine Seattle Washington USA

**Keywords:** golden angle spiral, in vivo blood T_1_, inversion recovery, k‐t sparse SENSE, single‐shot imaging

## Abstract

**Purpose:**

Blood T_1_ is a key parameter for hemodynamic quantification in both non‐contrast‐ and contrast‐enhanced imaging. Individual vessel T_1_ has been measured using a modified Look–Locker scheme with multi‐shot EPI or FLASH in high spatial resolution, requiring ∼1 min. Here, by exploiting the temporal sparsity from the excessive number of inversion delays, we apply Golden Angle rotated Spiral k‐t Sparse Parallel imaging (GASSP) to enable blood T_1_ measurement in a single shot of 10 s.

**Methods:**

The pulse sequence with single‐shot GASSP reconstruction was developed for T_1_ measurement from the internal jugular vein (IJV) with 1 × 1 mm^2^ in‐plane resolution. On nine healthy volunteers, the single‐shot GASSP was compared to the segmented EPI readout and was repeated to assess its intra‐scan reproducibility. Another experiment was performed on three patients, during which the 10 s GASSP was obtained at different time points prior to and following the Gadolinium (Gd) administration to assess dynamic changes in blood T_1_.

**Results:**

The blood T_1_ values measured with the highly undersampled GASSP method were strongly correlated (*r* = 0.83) with those using the multi‐shot EPI readout and exhibited high reproducibility (*r* = 0.88) within the session. The baseline IJV T_1_ values measured were 1700–2000 ms. Following the Gd injection, the T_1_ values of IJVs gradually recovered from ∼300–400 to ∼500 ms within 10–15 min.

**Conclusion:**

The feasibility of an ultrafast blood T_1_ measurement was demonstrated with high spatial resolution in a single shot of 10 s, applicable to both pre‐ and post‐contrast conditions.

## 1 Introduction

Blood T_1_ value is an essential parameter for quantifying hemodynamic measures such as cerebral blood flow (CBF) [1, 2, 3, 4, 5, 6] and cerebral blood volume (CBV) [7, 8, 9, 10, 11, 12] in non‐contrast‐enhanced MRI and has a critical impact on the arterial input function [13, 14, 15, 16, 17] in contrast‐enhanced imaging. T_1_ of blood is influenced by multiple factors [18, 19, 20, 21, 22, 23], including hematocrit, oxygenation, blood composition, contrast agent concentration, and magnetic field strength; therefore, making individual determination preferable.

Fast MR sequences have been developed to obtain in vivo blood T_1_, exploiting the continuous blood inflow, with a modified Look–Locker scheme in which a spatially non‐selective inversion pulse is followed by sequential slice‐selective 90° excitation and readouts at varying inversion time (TI). This approach was first applied in the 1990s to assess the glomerular filtration rate (GFR) by quantifying the concentration of contrast agent entering through the renal artery and exiting through the renal vein [24, 25]. The original implementation employed traditional gradient echo readouts, achieving limited in‐plane spatial resolution (around 2.0 mm in‐plane) and requiring long scan time (4 min) [24]. In the early 2010s, to facilitate CBF quantification using arterial spin labeling (ASL), the T_1_ estimation on moving blood was performed on the sagittal sinus [26, 27, 28, 29], internal jugular vein (IJV) [30], and internal carotid artery (ICA) [31]. For the readout: segmented balanced SSFP [26] and single‐shot EPI [27, 28, 29] were both applied, but with low spatial resolution (about 1.5 mm in‐plane) and thus high partial volume effect; alternatively, both segmented EPI [30] and turbo FLASH [31] with flow compensation achieved high resolution (∼1.0 mm in‐plane) with a scan time of approximately 1 min.

Given that the region of interest is confined to a small vessel and the rest of the tissue background is largely saturated, the excessive number of TIs (> 30) leads to high temporal sparsity. Golden Angle rotated Spiral k‐t Sparse Parallel imaging (GASSP) has been proposed as a promising solution to exploit this sparsity and has been utilized to achieve highly accelerated coronary images with high spatiotemporal resolution [32, 33, 34]. In addition, the center‐out spiral trajectory offers inherent robustness against flow artifacts for pulsatile blood signals.

In this study, we extended the GASSP framework to achieve high‐resolution (1.0 mm in‐plane), single‐shot blood T_1_ measurement sequence within about 10 s for efficient and accurate T_1_ quantification in the IJV. The proposed sequence was compared to the slower multi‐shot EPI readout with similar spatiotemporal resolution, and its reproducibility was evaluated. Additionally, GASSP was employed to monitor T_1_ changes during patient scans with gadolinium (GD) administration.

## 2 Methods

### 2.1 Pulse Sequence

**FIGURE 1:**
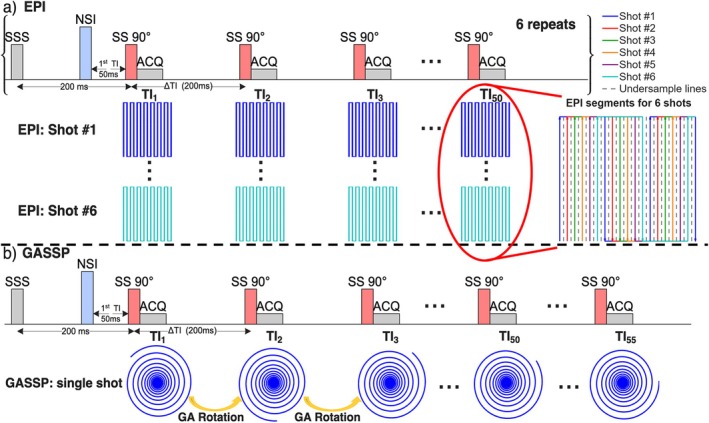
Pulse sequences for ultrafast T_1_ measurement of flowing blood using (a) previously implemented EPI and (b) newly proposed golden‐angle spiral projection (GASSP) sampling strategy. A non‐selective inversion (NSI) pulse is followed by (a) 50 or (b) 55 slice‐selective (SS) 90° excitation pulses spaced at 200 ms intervals (ΔTI) within a single TR. A slice‐selective saturation (SSS) pulse is applied 200 ms before the first SS excitation to suppress background signal from static tissues. Image acquisition (ACQ) is performed after each SS pulse using either a 6‐shot segmented EPI or a single‐shot GASSP readout. Panels below the pulse sequences illustrate the k‐space sampling patterns at ACQ for each method. For EPI, k‐space is segmented across six shots with interleaved TI sampling. The red circle demonstrates the complete EPI sampling pattern, where each color corresponds to one of the six EPI shots, and arrows indicate acquisition direction. Dashed gray lines represent the pseudo fully sampled trajectory, as the EPI acquisition uses a reduction factor of 2. For GASSP, a golden‐angle rotation is applied between 55 successive acquisitions in one single shot. The last 5 TI samplings were added to mitigate artifacts in the GASSP‐reconstructed images.

The pulse sequences for blood T_1_ measurement used in this study are illustrated in Figure 1, with an adiabatic non‐selective inversion (NSI) pulse followed by image acquisitions at 50 successive TIs, starting from 1st TI = 50 ms (to avoid eddy current effects [30]) with an increment of ΔTI = 200 ms. The NSI pulse was performed using a 13.3 ms hyperbolic secant pulse with a peak B1 power of 13.5 μT and a bandwidth of 1202 Hz. The image acquisition is performed after each 90° slice selective excitation pulse using either a 6‐shot segmented EPI (60 s) (Figure 1a) or a single‐shot GASSP (10 s) readout (Figure 1b).

The EPI readout with SENSE acceleration (reduction factor *R* = 2) was used. As shown in Figure 1a, k‐space was acquired with six shots (segments) with 50 TIs for an FOV of 200 (AP) × 160 (RL) mm^2^ and a resolution of 1.05 × 1.14 mm^2^. Identical k‐space patterns were applied to all 50 TIs in each shot. The red circle in Figure 1a demonstrates how the 6 k‐space segments were acquired in different shots for each TI with colored segments, whereas the gray dashed lines in EPI k‐space segments indicate the missing sampling trajectories for the 2‐fold SENSE acceleration. The EPI readout duration was 15 ms with a TE of 12 ms and an EPI factor of 11.

In contrast, GASSP acquired k‐space data in a single shot, with only one spiral arm sampled at each TI, rotated by the golden angle (GA = 137.51°) between consecutive TIs as illustrated in Figure 1b. A variable density undersampling pattern [35, 36] was applied to the spiral readout: the central 15% of k‐space was fully sampled; the outer 50% was undersampled by a factor of 5; and the reduction factor increased linearly with the radius in the intermediate region. Imaging parameters include FOV = 180 × 180 mm^2^, resolution = 1.0 × 1.0 mm^2^, TE = 2.1 ms, and 12 ms readout duration. Five additional TIs were acquired for GASSP (Figure 1b, a total of 55 TIs) to avoid artifacts induced by boundary conditions of the sparsity transform along the temporal dimension, which will be detailed in the image reconstruction section below.

All spins draining into the imaging slice are assumed to have experienced the NSI pulse. As blood velocities in the IJVs of normal adults are typically under 40 cm/s, and with the slice thickness (THK) set to 5 mm, the condition ∆TI>THK/v is satisfied [30]. This ensures that venous blood magnetizations saturated by the previous excitation pulses would be refreshed by upstream spins after 200 ms delays before the following excitations. While flowing blood was continually replaced by fully inverted spins, static tissue remaining within the imaging slice was continuously saturated by the 90° excitation pulses, with T_1_ recovery of the longitudinal signal during the 200 ms interval. A slice‐selective WET saturation pulse train (vendor provided, tailored with four 4.2‐ms numerically optimized “rest_2” pulses: 89°, 98°, 82°, 157°) [37, 38] covering the imaging slice was applied 150 ms prior to the NSI pulse to pre‐saturate static background signal and prevent high background signal at the first excitation. The WET saturation pulse was designed to suppress tissues within a wide T_1_ range of 400–4200 ms [38].

### 2.2 In Vivo Experiments

All experiments were performed on a 3 T Philips Ingenia scanner (Philips Healthcare, Best, The Netherlands) using a 32‐channel head‐only receive coil. Nine healthy volunteers (28 ± 8 years old, 4 females) and three patients with pretreated brain metastases (BM) were enrolled and all provided informed written consent in accordance with the Johns Hopkins Institutional Review Board.

As a part of the scan planning process, a 3D velocity‐selective MRA (VSMRA) survey image was obtained to quickly visualize IJVs and other major neck vessels. The VSMRA survey used a Fourier transform based velocity selective saturation (FT‐VSS) pulse train [12, 39, 40] with a cutoff velocity of 0.7 cm/s (9 segments, FT‐VSS pulse train duration = 80 ms, velocity sensitive gradients strength/duration/slope = 26 mTm^−1^/0.8 ms/0.2 ms). Acquisition parameters were TR/TE/readout = 17/2.9/9.5 ms (stack‐of‐spiral), FOV (axial) = 180 × 180 × 100 mm^3^, and a scan resolution of 1.5 × 1.5 × 2.0 mm^3^ (reconstructed to 0.75 mm in‐plane), acquisition time = 34 s.

Blood T_1_ measurements were conducted on a 5 mm slice positioned perpendicular to both IJVs slightly below the level of the sigmoid sinus (Figure S1). The GASSP acquisition was compared to the EPI acquisition and then repeated within the session to assess intra‐scan reproducibility for all nine subjects.

Experiments were also performed on the BM patients to evaluate the performance of the GASSP method under dynamic conditions in which blood T_1_ changes over time. To this end, the 10 s single‐shot GASSP acquisition was repeated at different time points before and after the Gd injection.

### 2.3 Image Reconstruction

EPI data were reconstructed using vendor‐provided online reconstruction. GASSP was implemented in MATLAB (MathWorks Inc., Natick, MA, USA) using non‐Cartesian SENSE data fidelity regularized by the Total Variation (TV) sparsity transform along the TI dimension. Reconstruction solves the following cost function:

$$I=\underset{I}{\text{argmin}}\left({\left\|k-SFI\right\|}_{2}^{2}+\lambda {\left\|TV_{\mathrm{TI}}\left(I\right)\right\|}_{1}\right),$$

where I represents the reconstructed image, k denotes the undersampled k‐space data, S is the coil sensitivity map, and F is the non‐uniform Fourier transform for spiral acquisition. $V_{\mathrm{TI}}\left(\cdot \right)$ is the TV operator along the TI dimension, and λ = 0.1 is the weight for the sparsity regularization. Image reconstruction was performed using an iterative conjugate gradient algorithm.

To mitigate potential image artifacts and signal loss at the first and last few TIs (shown in the Result section), 5 additional TIs (51st–55th, 10 050–10 850 ms) were appended to the end of the series. The 4 images of the 52nd–55th TIs are also prepended to the beginning of the series (before the first TI, periodic padding) with their k‐space signal inverted, resulting in a total of 59 frames. Moreover, the rotation angle between the 55th TI (137.51° × 54 = 7425.54°) and the first TI (0°), when taken modulo 360°, was 134.46°, which was close to the GA rotation (137.51°). These additional frames were only incorporated for GASSP image reconstruction and were excluded from T_1_ fitting. After GASSP reconstruction, images were deblurred using the deconvolution process [41] with kernels calculated from the spiral trajectory and a B0 map automatically performed by the scanner for the VSMRA survey image.

### 2.4 Data Analysis

All data processing was performed in MATLAB. Segmentation of the vessel areas was conducted semi‐automatically. A seed pixel was manually selected within the IJV on the first phase, and a region of interest (ROI) was then generated using the full‐width at half‐maximum (FWHM) method. To avoid partial volume effects, the FWHM threshold was set to 0.7. To prevent potential motion‐induced misalignment in the single‐shot GASSP image series, a motion registration was performed before averaging to a 20 × 20 mm^2^ area surrounding the target vessel, with frame‐to‐frame rigid image registrations between consecutive TIs using regular step gradient descent optimization. This motion tracking was not applied to images acquired with multi‐shot EPI readout, because each image was a combination of six segments with potential inter‐segment motion‐induced misalignment. Blood T_1_s were estimated by nonlinear least‐squares fitting of the average signal within the vessel ROIs using the T_1_ relaxation model:

$$M=M^{0}+\left(M_{\text{init}}-M^{0}\right)e^{-\mathrm{TI}/T_{1}},$$

where M is the measured signal, $M_{\text{init}}$ is the initial signal immediately after inversion, and $M^{0}$ represents the equilibrium signal. **T**_**1**_ values were fitted for bilateral IJVs of all subjects and for bilateral internal carotid arteries (ICAs) of the BM patient. For *N* = 9 normal subjects, linear regressions and Bland–Altman plots were performed to compare between the T_1_ values measured with EPI and GASSP acquisitions, as well as to assess the intra‐scan reproducibility of GASSP. For the BM patient, changes in blood T_1_ of the IJVs and ICAs were analyzed during the patient scan before and following Gd injection using the GASSP protocol.

## 3 Results

**FIGURE 2:**
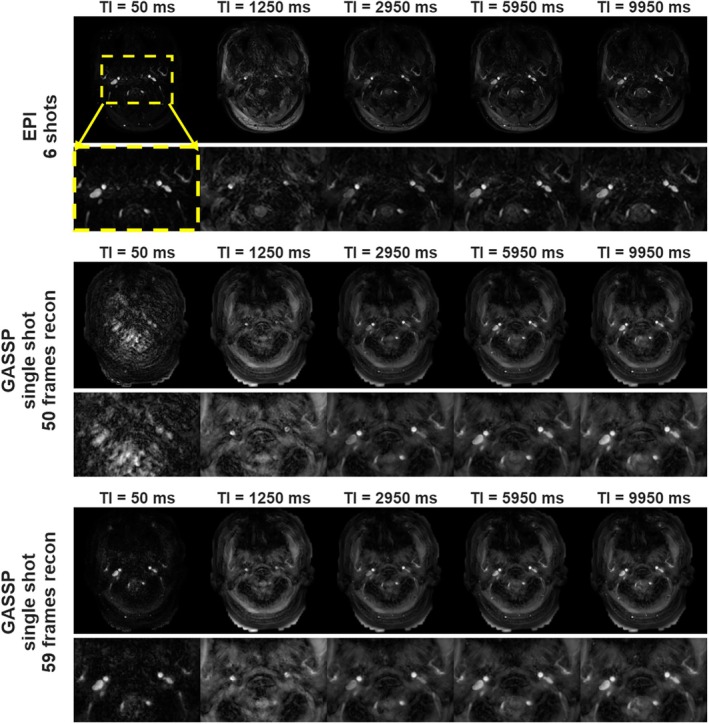
Comparison of image quality among traditional 6‐shot EPI (top panel), single‐shot GASSP with 50 frames reconstruction (middle panel), and single‐shot GASSP with 59 frames reconstruction (bottom panel) at selected inversion times (TI = 50/1250/2950/5950/9950 ms) on a 28‐year old male subject. The top row of each panel displays the full FOV, while the bottom row shows a zoomed‐in ROI, as indicated by the yellow dashed box in the top panel. The images show bilateral ICAs and IJVs in an axial slice, highlighting both venous and arterial structures. When the GASSP data were reconstructed using both appended and prepended 9 frames, image artifacts and signal loss were eliminated in the original 50 frames.

**FIGURE 3:**
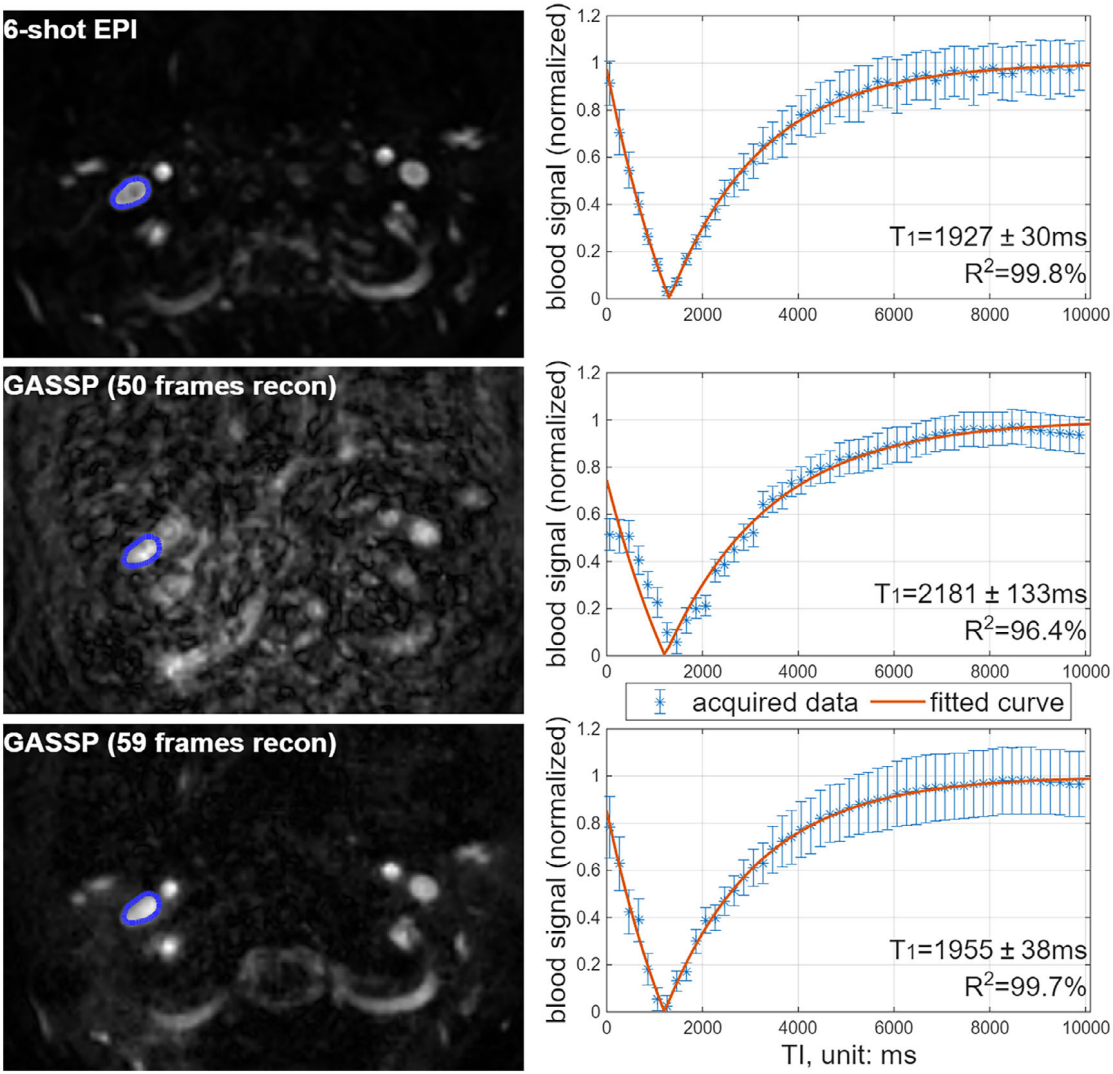
Representative reconstructed images at TI = 50 ms and the blood T_1_ fitting curves of the right IJV with EPI (top panel), GASSP with 50 frames construction (middle panel) acquisitions, and GASSP with 59 frames construction (bottom panel) acquisitions, on a 20‐year old female subject. Contours of the right IJV are drawn on the images as blue lines. Relative to the EPI acquisition (top panel), the 50‐frame GASSP reconstruction showed artifacts that underestimated blood signal at early and late TIs, leading to overestimated blood T_1_ and reduced *R* ^2^ (middle panel). Adding five extra TIs (10 050–10 850 ms) at the end and four pseudo–TI = 0 frames at the beginning yielded consistent T_1_ values and high *R* ^2^ (> 99%) in the fitting (bottom panel).

Figure 2 compares images from a conventional 6‐shot, 2‐fold undersampled EPI and single‐shot, 12‐fold undersampled GASSP acquisitions of one subject at selected TIs. In the GASSP‐reconstructed images, when only the 50 acquired TIs (frames) identical to the EPI acquisition were used, image artifacts and signal loss appeared in the vessel ROIs at the beginning and end of the TI series (Figure 2). These artifacts led to overestimated blood T_1_ values and reduced *R* ^2^ in the least square fitting (Figure 3). When the GASSP data were reconstructed using the nine additional frames, image artifacts and signal loss were minimized compared to using the original 50 frames (Figure 2). The resulting T_1_ maps yielded fitted T_1_ values in IJVs with high *R* ^2^ (> 99%) are consistent with those from the EPI acquisition, in contrast to the GASSP reconstruction using only 50 frames (Figure 3). Unless otherwise specified, the term “GASSP” in the following sections denotes the 59‐frame reconstruction.

**FIGURE 4:**
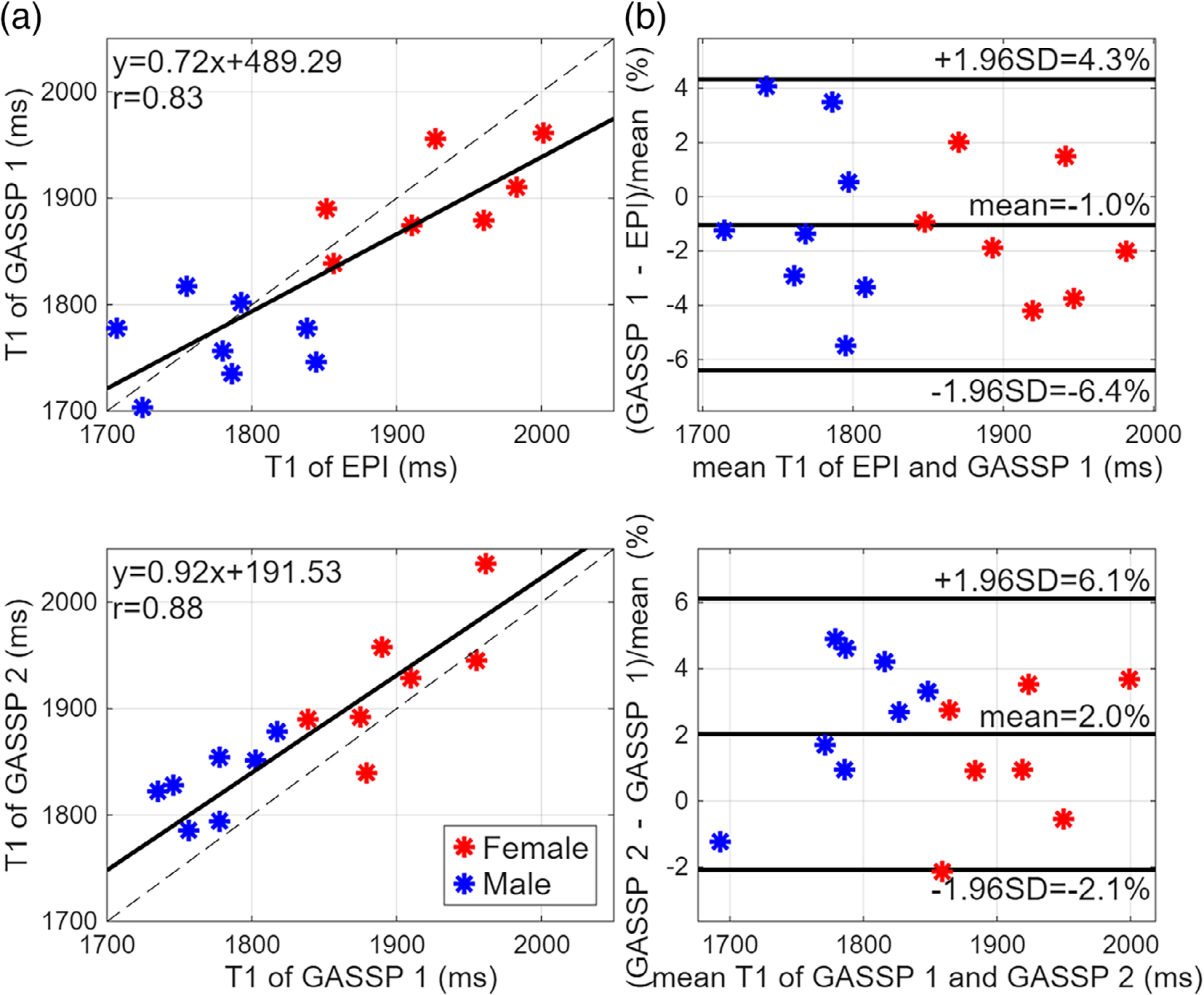
Scatter plot with linear regression (left) and Bland–Altman plots (right) of IJV T_1_ values obtained (a) from EPI scans versus the proposed GASSP scans and (b) from the two GASSP scans for repeatability comparison.

Statistical results of the IJV T_1_s from all 9 subjects are demonstrated in Figure 4. 3 out of 18 IJVs (bilateral from 9 subjects) were excluded due to low fitting confidence (95% confidence interval of least square fitting ≥ 100 ms), likely related to their small vessel size. In Figure 4a, the T_1_ values estimated with the proposed single‐shot GASSP acquisition were compared to the conventional 6‐shot EPI acquisition. The scatter plot (top left) shows a strong linear correlation (*r* = 0.83), and the Bland–Altman plot (top right) indicates a small T_1_ bias of 1% with randomly distributed errors, demonstrating high consistency between the two methods. The intra‐scan reproducibility of the GASSP technique is demonstrated in Figure 4b. The corresponding scatter plot demonstrates a strong correlation (*r* = 0.88) between repeated T_1_ measurements, and the Bland–Altman plot indicates a small mean bias of −2%. These findings confirm high intra‐scan reproducibility of the GASSP protocol. Additionally, females (red markers) consistently showed longer venous T_1_ values than males (blue markers), reflecting their expected hematocrit differences [30, 31, 42].

**FIGURE 5:**
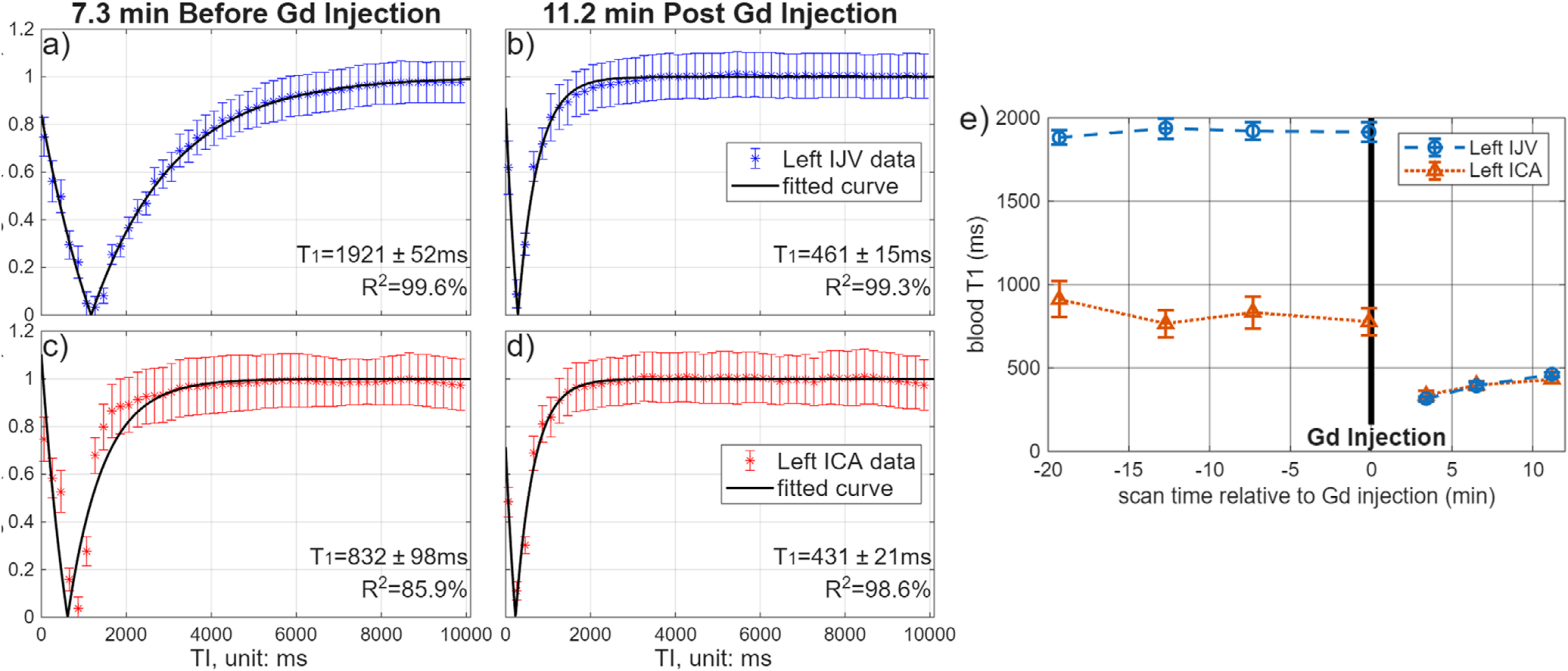
Blood T_1_ fitting curves of (a, b) the left IJV and (c, d) left ICA of a 34‐year‐old female patient with brain metastases measured with GASSP before and after the Gadolinium (Gd) injection. (e) Changes of blood T_1_ values in left IJVs and left ICAs measured by the GASSP protocol at four time points before the Gd injection (19, 13, 7 min, and 9 s), and three time points (3, 7, 11 min) after the Gd injection.

As shown in Figures 5 and S2, prior to Gd administration, T_1_ values were markedly longer in the IJVs (1900–2100 ms) than in the ICAs (800–1000 ms), as expected and further explained in Section 4; following Gd injection, T_1_ values in both vessels dropped significantly and converged to 300–400 ms, then gradually increased to about 500 ms within 10–15 min, indicative of contrast agent wash‐out.

## 4 Discussion

In this work, an ultrafast blood T_1_ measurement scheme was demonstrated using the GASSP technique for image acceleration. The IJV blood T_1_ values obtained using the proposed technique were comparable to those from the previous multi‐shot EPI (Figure 3) acquisition and demonstrated high intra‐scan reproducibility (Figure 4). Furthermore, with a 10‐s acquisition, arterial and venous blood T_1_ changes were tracked with high temporal precision during Gd wash‐in and wash‐out (Figure 5).

Compared to the original modified Look–Locker pulse sequence, we introduced a pre‐saturation of the imaging slice prior to the NSI pulse. This modification was necessary to suppress the static background signal from the first excitation. Without pre‐saturation, the initial excitation would have the near‐equilibrium background signal with negative polarity immediately after the NSI pulse, while subsequent excitations would yield saturation recovery every 200 ms. This large signal variation across TIs would compromise the sparsity of the TV transform in the TI domain and hinder the convergence of the GASSP reconstruction. Incorporating a pre‐saturation pulse mitigated this issue by ensuring a consistent background signal level and maintaining the temporal sparsity essential for successful reconstruction.

The proposed method employs spiral acquisition to achieve high acquisition efficiency, which also provides improved incoherence compared with Cartesian sampling, thereby increasing the feasibility of compressed sensing (CS)‐based reconstruction [43]. Previous spiral‐based parametric mapping methods have exploited the spatial sparsity using individual CS‐SENSE reconstructions [44, 45], or temporal sparsity for (pseudo) golden angle rotated spirals with regularization strategies such as temporal total variation, low‐rank constraints, parametric models, or more advanced approaches such as magnetic resonance fingerprinting [34, 46, 47, 48]. The proposed method adopts temporal total variation for image reconstruction, motivated by several factors: (1) the high sparsity of the temporal total variation transform enabled by background suppression and substantial temporal redundancy across abundant temporal frames, (2) improved accuracy for vessel segmentation by avoiding partial volume effects induced by spatial sparsity constraints, and (3) the simplicity of implementation.

Using only 50 TIs (matching the EPI acquisition) in the GASSP reconstruction caused image artifacts around vessel ROIs at the beginning and end of the TI series (Figure 2). The likely cause was the TV transform's assumption of zero gradient at the temporal boundaries, even though the vessel signal continued to change dynamically. In the proposed approach, appending 5 additional TIs allowed the blood signal to approach equilibrium, and prepending images from the last 4 appended TIs before the first sampled TI effectively simulated the expected signal at TI = 0 ms. As a result, the background signal remained largely saturated, rendering the difference between the prepended frames and the first sampled TI sparse in the TV domain. This configuration provided both sufficient temporal sparsity and incoherent aliasing, enabling stable GASSP reconstruction.

The current blood T_1_ measurement protocol was optimized for the IJV. The required global uniform inversion was insufficient for the arterial blood magnetization in the chest region, where large B_0_ and B_1_ inhomogeneities are present [9]. Consequently, the ICA T_1_ values appeared much lower than the corresponding IJV values (Figures 5 and S2). After Gd injection, the blood T_1_ shortened to 300–400 ms, which reduced the effect of rapidly recovered arterial blood originated from the incompletely inverted chest region, resulting in comparable post‐contrast T_1_ values between the ICA and IJV. The proposed protocol could be adapted for ICA pre‐contrast by repositioning the isocenter to the clavicle level and employing an inversion pulse with a broader bandwidth and adequate adiabaticity to tolerate the large B_0_/B_1_ field inhomogeneity [31]. Alternatively, arterial blood T_1_ could be inferred from the measured IJV T_1_ by adding a correction term accounting for hematocrit and oxygenation. In our previous 3 T study [31], ICA T_1_ values were found 56 ± 35 ms longer than IJV T_1_ in 13 healthy volunteers (hematocrit = 0.42 ± 0.04). It was postulated that this arterial–venous T_1_ difference (∼56 ms) was smaller than the theoretical estimate [23] (∼158 ms) due to partial contributions from capillary blood [31]. Importantly, even a 100 ms overestimation would introduce only about a 5% error for an arterial T_1_ of 1900 ms, which is negligible. Thus, using IJV T_1_ rather than ICA T_1_ circumvents technical challenges and is more practical for integration into standard neuroimaging protocols.

Rapid and precise blood T_1_ measurements following contrast injection would allow more accurate, subject‐specific characterization of contrast agent concentration kinetics. Its much faster recovery to equilibrium may require sampling fewer than half of the TIs, potentially reducing the scan time to only a few seconds. Beyond hemodynamic mapping, this method can also be used in studies monitoring CSF circulation and clearance after intravenous Gd injection. In addition to neuroimaging, this method also holds potential for optimization toward [49, 50, 51] more accurate quantification of myocardial infarction based on late Gd enhancement [52, 53].

## 5 Conclusions

We have demonstrated the feasibility of an ultrafast IJV blood T_1_ measurement technique with 1 × 1 mm^2^ in‐plane resolution in a single shot of 10 s, by taking advantage of temporal sparsity of the data using GASSP reconstruction. T_1_ measured with GASSP was highly correlated with the previous method and exhibited high reproducibility. With its extremely short acquisition time, this technique has the potential to facilitate precise, subject‐specific characterization of arterial input functions applicable to both non‐contrast and contrast‐enhanced MRI methods.

## Funding

Grant support from NIH R01 HL144751; NIH RF1 AG082243; NIH R01 CA282928; NIH R01 NS139369; NIH P41 EB031771; NIH K25 AG083114.

## Supporting information


**Figure S1:** (a) One sagittal slice of the survey image. (b) and (c) sagittal and coronal maximum intensity projection (MIP) images of the 3D velocity‐selective MR angiography (VSMRA) scan. (d) VSMRA axial image for selected slice, showing IJVs. The orange line indicates the imaging slice selected for T_1_ measurements, chosen perpendicular to the internal jugular veins (IJVs).
**Figure S2:**. Changes of blood T_1_ values in IJVs and ICAs measured by the GASSP protocol during scans of two patients with brain metastases (a) an 83‐year old male; (b) a 72‐year old female. (a) Measurements were acquired at one time point before the Gd injection (−18 min), and three time points (3, 6, 11 min) after the Gd injection. (b) Measurements were acquired at two time points before the Gd injection (−7, −1 min), and three time points (2, 5, 10, 14, 17 min) after the Gd injection.

## Data Availability

The data that support the findings of this study are available from the corresponding author upon reasonable request.
