# Supplementary material for: Ultrafast Blood T1 Measurement Using Golden Angle Rotated Spiral k‐t Sparse Parallel Imaging (GASSP): Evaluations in Both Pre‐ and Post‐Contrast Conditions
Source: Magn Reson Med. 2026 Feb 6;95(6):3351–9. doi: 10.1002/mrm.70286 (PMC13049252; doi:10.1002/mrm.70286)

**Figure S1**: **a**): one sagittal slice of the survey image. **b**) and **c**): sagittal and coronal maximum intensity projection (MIP) images of the 3D velocity-selective MR angiography (VSMRA) scan. **d**): VSMRA axial image for selected slice, showing IJVs. The orange line indicates the imaging slice selected for T_1_ measurements, chosen perpendicular to the internal jugular veins (IJVs).

**
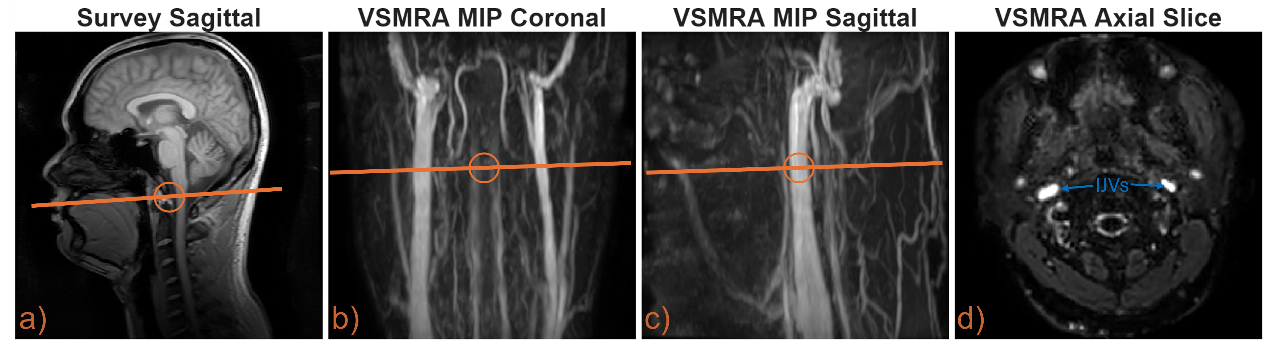
**

**Figure S2**: Changes of blood T_1_ values in IJVs and ICAs measured by the GASSP protocol during scans of two patients with brain metastases (a) an 83-yo male; (b) a 72-yo female. **a**): Measurements were acquired at one time point before the Gd injection (-18 min), and three time points (3 min, 6 min, 11 min) after the Gd injection. **b**): Measurements were acquired at two time points before the Gd injection (-7 min, -1 min), and three time points (2 min, 5 min, 10 min, 14 min, 17 min) after the Gd injection.


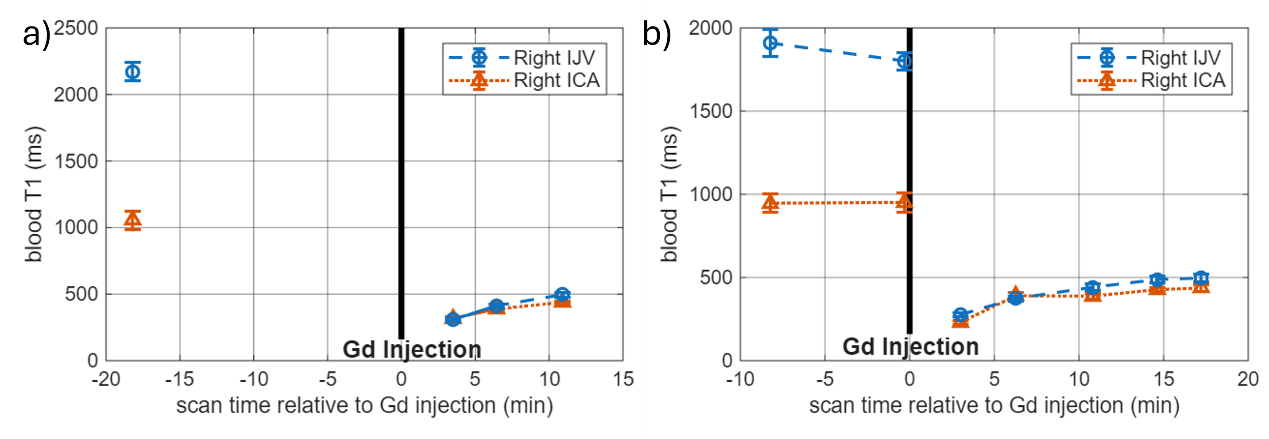

Supplement: Supplementary file 1 — Figure S1: (a) One sagittal slice of the survey image. (b) and (c) sagittal and coronal maximum intensity projection (MIP) images of the 3D velocity‐selective MR angiography (VSMRA) scan. (d) VSMRA axial image for selected slice, showing IJVs. The orange line indicates the imaging slice selected for T1 measurements, chosen perpendicular to the internal jugular veins (IJVs). Figure S2:. Changes of blood T1 values in IJVs and ICAs measured by the GASSP protocol during scans of two patients with brain metastases (a) an 83‐year old male; (b) a 72‐year old female. (a) Measurements were acquired at one time point before the Gd injection (−18 min), and three time points (3, 6, 11 min) after the Gd injection. (b) Measurements were acquired at two time points before the Gd injection (−7, −1 min), and three time points (2, 5, 10, 14, 17 min) after the Gd injection. [file MRM-95-3351-s001.docx]
